# Preservation of mouse ovarian tissue follicle morphology and ultra-structure after vitrifying in biotechnological protocols

**DOI:** 10.1186/s13048-015-0137-3

**Published:** 2015-03-06

**Authors:** Hamid Tayefi Nasrabadi, Maryam Gavami, Abolfazl Akbarzadeh, Rahim Beheshti, Daryosh Mohammadnejad, Ali Abedelahi

**Affiliations:** Department of Anatomical Sciences, Tabriz University of Medical Sciences, Tabriz, Iran; Department of Veterinary, Shabestar Branch, Islamic Azad Universit, Shabestar, Iran; Department of Medical Nanotechnology, Faculty of Advanced Medical Science, University of Medical Sciences, Tabriz, Iran; Drug Applied Research Center, Tabriz University of Medical Sciences, Tabriz, Iran

**Keywords:** Ovarian tissue, Cryopreservation, Vitrification, Ultrastracture

## Abstract

**Background:**

The aim of the present study was to characterize the morphological and ultrastractural of mouse ovarian tissue with different cryoprotectant solution.

**Objective:**

Aim of this study, is to demonstrae an improved convetional vitrification method on mouse ovarian tissue using different concentrations of ethylene glycol (EG) and/or dimetyl sulfoxide (DMSO) and EG.

**Materials and methods:**

Mouse ovarian tissue dissected and were randomly assigned to three groups: control, conventional vitrification (CV) and toxicity test. Then ovaries were vitrified by 5%, 10% EG or DMSO CV1-CV4, 5%, 10% EG plus DMSO CV5-CV6 and EG plus DMSO in climbing concentrations CV7. The effect of cryoprotectant solutions on ovarian tissue were evaluated by histological examination hematotoxillin & eosin stain, H&E, viability assessment trypan blue stain and ultrastructural analyses transmission electron microscopy, TEM.

**Results:**

Ovarian tissue frozen in CV7 solution showed a higher percentage of morphologically normal follicles or viable follicles than other cryoprotectant solutions P < 0.05. Ultrastructural analysis of ovarian tissue showed that less damage was observed in CV7 and it was very similar to the control group.

**Conclusion:**

Vitrification of ovarian tissue with optimal cryoprotectant solutions such as EG plus DMSO is the most effective for preserving the structural efficiency of ovarian follicles.

## Background

The cryopreservation of ovarian tissue is a suitable technique to reservation the fertility of women and young girls before undertaking chemotherapy or radiotherapy and high risk for premature and genetic mutations [1].

So many efforts have been made to progress cryopreservation conditions by simple and effective procedures “vitrification” [2-4] There are controversial results about the induction of apoptosis by vitrification [4,5] Some researchers indicated that vitrification resulted on structural damages of ovarian tissue vitrification [6,7]. In contrast, our previous results revealed that the organelles of granulosa cells and oocyte were well preserved in conventional vitrification.

Recently EG and DMSO have been used by numerous authors as cryoprotectants for cryopreservation of ovarian tissue [8,9]. Viable tissue has been obtained after conventional freezing with DMSO and EG [1]. Santos et al. reported that preantral follicle morphology was not affected after conventional vitrification [10]. However, the vitrification technique and cryoprotectants agents are major factors in the cryopreservation of ovarian tissue. Because of the main challenge is to find the optimum composition for a cryoprotectant solution having as little a biologic toxicity and damage for ovarian tissue.

## Objective

Scope of this article, is to demonstrae an improved convetional vitrification method on mouse ovarian tissue using different concentrations of EG and/or DMSO and EG.

## Materials and methods

### Animal and ovarian tissue

All material used in this study were buied from Sigma-Aldrich Co. Hamburg-Germany.

Five to six healthy week-old Balb/c female mice n = 64 were sourced from tabriz university of medical center agreement with the International Animal Care and Use Committee and accommodated in temperature standard condition 22 ± 2°C. Nutrition and water were freely accessible at all times 12 h light and 12 h dark.

### Vitrification and thawing

After dissecting of ovarian tissue, they were accidentally assigned to three groups: control, vitrification, toxicity test.

In this study, Vitrification solutions: 5% EG in Dulbecco’s phosphate- buffered saline (DPBS) with 0.5 M sucrose + 20% FBS group CV1, 10% EG in DPBS with 0.5 M sucrose + 20% FBS group CV2, 5% DMSO in DPBS with 0.5 M sucrose + 20% FBS group CV3, 10% DMSO in DPBS with 0.5 M sucrose + 20% FBS group CV4, 5% EG + 5% DMSO in DPBS with 0.5 M sucrose + 20% FBS group CV5, 10% EG + 10% DMSO in DPBS with 0.5 M sucrose + 20% FBS group CV6 and EG plus DMSO in ascending concentrations or equilibrated in solutions of CV 5 then vitrified in CV 6 group CV7.

After dehydration of ovaries they were place in a 1.5 ml plastic cryotubs with a least volume of the vitrification solutions and were placed on nitrogen gas for 10 sec and then were placed into liquid nitrogen and kept for one week.

For thawing, vitrified ovaries were warmed in room temperature for 10 sec and then placed in 25°C water bath for 10 sec and then supplemented with descending concentration of sucrose1, 0.5 and 0.25 M and DPBS at room temperature for 10 minutes. For toxicity test, the ovarian tissue exposed all stages of vitrification and warming procedure except plunging in liquid nitrogen.

### Isolation of ovarian follicles and trypan blue staining for viability assessment

Ovarian follicles were mechanically isolated by 29-gauge needles under a stereomicroscope and then transferred to new microdroplets 20 μl and covered with mineral oil.

In each group, follicles that have layers of membrane-enclosed granulosa cells and centrally located oocyte were examined and then samples stained in a Petri dish with 0.4% final trypan blue. The follicular viability and the follicular stage were assessed under an inverted microscope at × 400 magnification. Degenerated follicles were stained blue, and live ones were unstained.

### Histological protocol and morphologic evaluation

For morphological and histological assessment, ovaries in control, toxicity test, and vitrified group (n = 5 for each group) were dehydrated in ethanol, clarified with xylene, embedded in paraffin wax and sectioned at 5 mm thickness. The sections were stained with hematoxylin and eosin HE and examined under light microscope. Follicular quality was evaluated and follicles were classified as normal or degenerate.

Ovarian follicles were classified according to their developmental stage as primordial one layer of flattened or flattened–cuboidal granulosa cells around the oocyte, primary one layer of cuboidal granulosa cells around the oocyte and secondary follicles two or more layers of cuboidal granulosa cells around the oocyte.

### Ultrastractural evaluation

All samples were randomly collected n = 3 from each groups after equilibration in medium for 30 minutes and fixed in 2.5% glutaraldehyde in phosphate-buffered saline pH 7.4 for 2 h and then treated 1% osmium tetroxide at 4°C for 2 h. Post-fixed specimens were dehydrated gradually in ethanol solutions of different concentration and then samples were located in propylene oxide and embedded in Epon 812. The sections (60 nm) were placed on copper grids, and stained with uranyl acetate and Reynolds’ lead citrate.

Then was investigated the the integrity of the cytoplasmic membrane and the mitochondrial cristae and nuclear membrane and cytoplasmic organelles of granulosa cells and oocyte.

### Statistical analaysis

Statistical analysis for survival rate of follicles in all goups were carried out using analysis of variance ANOVA and Tukey’s test with SPSS software 16.0 . A value of P < 0.05 was considered statistically significant.

## Results

### Survival rates of follicles derived by isolation from fresh and vitrified-thawed ovaries

Percentages of viable ovarian follicles derived from non-vitrified and vitrifed ovaries after trypan blue staining is presented in Table 1.

**Table 1:** **Number and percentage of intact (Int) and degenerated (Deg) follicles of various stages isolated from vitrified and non-vitrified ovarian tissue after Trypan blue staining**

**Group**	**Total number of follicles**	**Number of primordial follicles (%)**				**Number of primary follicles (%)**				**Number of secondary follicles (%)**			
		**Total**	**Int**	**Deg**	**Stat**	**Total**	**Int**	**Deg**	**Stat**	**Total**	**Int**	**Deg**	**Stat**
Control	259	73	72 (99)	1 (1)	-	91	88 (98)	2 (2)	-	95	93 (98)	2 (2)	-
CV1	232	75	69 (93)	6 (7)	-	84	73(86)	11 (14)	a, b	73	62 (83)	11 (17)	a, b
CV2	275	84	79 (94)	5 (6)	-	96	85 (88)	11 (12)	a, b	95	81 (85)	14 (15)	a, b
CV3	257	81	5 (92)	6 (8)	-	89	79 (89)	10 (11)	a, b	87	75 (85)	12 (15)	a, b
CV4	243	69	63 (91)	6 (9)	-	100	90 (90)	10 (10)	a	74	62 (82)	12 (18)	a, b
CV5	233	88	83 (95)	5 (5)	-	83	78 (94)	5 (6)	-	62	55 (88)	7 (12)	a, b
CV6	227	68	65 (95)	3 (5)	-	86	82 (95)	4 (5)	-	73	68 (93)	5 (7)	-
CV7	266	85	82 (97)	3 (3)	-	90	87 (97)	3 (3)	-	91	89 (98)	2 (2)	-

^a^P < 0.05 versus non-vitrified control group.

^b^P < 0.05 versus vitrified tissue in CV7 group.

CV, Conventional vitrification.

There was no significant effect of the cryoprotectant type on the viability of primordial follicles P > 0.05. Furthermore, when double cryoprotectant EG plus DMSO was used, primary and secondary follicles had higher viability than that achieved from single cryoprotectant P < 0.05. When data of all treatments were combined, the survival rate of the secondary follicles was significantly reduced in DMSO group 83% and were similar in EG 84% and EG + DMSO 85% groups. However, when ovarian tissue vitrified in ascending concentrations of EG plus DMSO CV7 group, increased the survival of secondary follicles P < 0.001.

### Histological analysis of ovarian tissue

For histological analysis, ovarian tissue follicles were classified as primordial, primary or secondary according to the shape and number of layers of granulosa cells. The normality and morphology percentage of follicles in various developmental stages after treatment with different vitrification methods and non-frozen controls are presented in Table 2.

**Table 2:** **Number and percentage of intact (Int) and degenerated (Deg) follicles of various stages evaluated in vitrified and non-vitrified ovarian tissue after Hematoxylin- eosin staining**

**Group**	**Total number of follicles**	**Number of primordial follicles (%)**				**Number of primary follicles (%)**				**Number of secondary follicles (%)**			
		**Total**	**Int**	**Deg**	**Stat**	**Total**	**Int**	**Deg**	**Stat**	**Total**	**Int**	**Deg**	**Stat**
Control	329	156	154 (99)	2 (1)	-	103	101 (98)	2 (2)	-	70	67 (96)	3 (4)	-
CV1	308	142	136 (96)	6 (4)	-	99	90 (91)	9 (9)	a	67	38 (59)	29 (41)	a, b
CV2	310	148	142 (96)	6 (4)	-	88	81 (92)	7 (8)	-	74	48 (64)	26 (36)	a, b
CV3	299	140	134 (96)	6( 4)	-	90	81 (90)	9 (10)	-	69	42 (60)	27 (40)	a, b
CV4	309	141	135 (96)	6 (4)	-	94	85 (91)	9 (9)	a	74	48 (64)	26 (36)	a, b
CV5	338	165	159 (97)	6 (3)	-	97	93 (96)	4 (4)	-	76	59 (77)	17 (23)	a, b
CV6	316	152	147 (97)	5 (3)	-	98	94 (96)	4 (4)	-	66	52 (79)	14 (21)	a
CV7	337	162	158 (98)	3 (2)	-	104	101 (97)	3 (3)	-	71	67 (95)	4 (5)	-

^a^P < 0.05 versus non-vitrified control group.

^b^P < 0.05 versus vitrified tissue in CV7 group.

CV, Conventional vitrification.

#### Morphology of primordial follicles after vitrification-thawing

A total of 1206 primordial follicles (approximately 150 follicles in each group) were examined for morphological analysis (Table 2). The percentage of morphologically normal follicles in fresh ovarian tissue (98%) was not significantly different (P > 0.05) from that observed after exposure to DMSO (96%), EG (95%), or DMSO + EG (97%).

**Figure 1:**
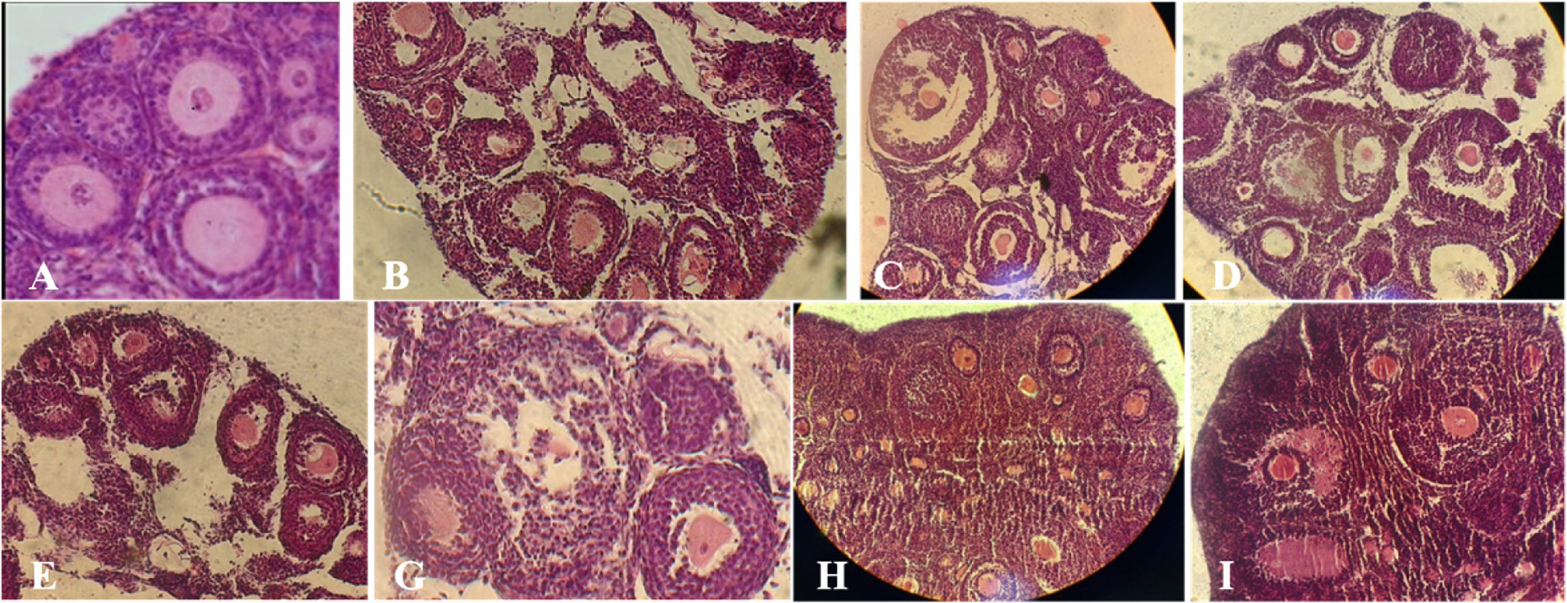
**Morphological images of the mouse ovarian tissue. A)** non-frozen, **B)** CV1 group that vitrified alone in 5% EG, **C)** CV2 group that vitrified alone in 10% EG, **D)** CV3 group that vitrified alone in 5% DMSO, **E)** CV4 group that vitrified alone in 10% DMSO, **G)** CV5 group that vitrified in 5% EG + 5% DMSO, **H)** CV6 group that vitrified in 10% EG + 10% DMSO and **I)** CV7 group that equilibrated in CV5 and then CV6 and finally vitrified in CV6 concentration.

Primordial follicles consisted of an oocyte surrounded by one layer of flattened or flattened cuboidal granulosa cells (Figure 1A). In these follicles, cuboidal granulosa cells, when present, were usually located on opposite poles of the follicles, giving them an elongated appearance.

#### Morphology of primary follicles after vitrification-thawing

A total of 773 primary follicles (96 per treatment) were histologically analyzed in fresh and vitrified ovaries (Table 2). The percentage of normal primary follicles in non vitrified ovaries (control) was higher than CV1 and CV3 group. In the other hand, when ovarian tissues had been frozen/thawed in the presence of EG or DMSO at a concentration of 5% resulted in a significant reduction in the percentage of normal primary follicles (*P* < 0.05).

In primary follicles, the oocyte was surrounded by one layer of cuboidal granulosa cells (Figure 1A). The morphology of primary follicles was well preserved in the control and ovaries that vitrified in double cryoprotectant. The cytoplasm and oocytes of follicles was clear and normal. Also the granulosa cells were intact and firmly attached to the related basement membranes (Figure 1G-I).

#### Morphology of secondary follicles after vitrification-thawing

In total of 567 secondary follicles (70 per treatment) were histologically examined in non-vitrified and vitrified groups (Table 2). The percentage of normal secondary follicles were significantly higher (P < 0.001) in ovarian tissue vitrified in a stepwise increasing double cryoprotectants (95%) than that of follicles that had been cryopreserved in EG (61%) DMSO (62%), 5% EG + DMSO (77%) and 10% EG + DMSO (79%). A very low rate of degeneration was observed in non-vitrified ovarian tissue and CV7 groups (3.66% vs 4.47%).

**Figure 2:**
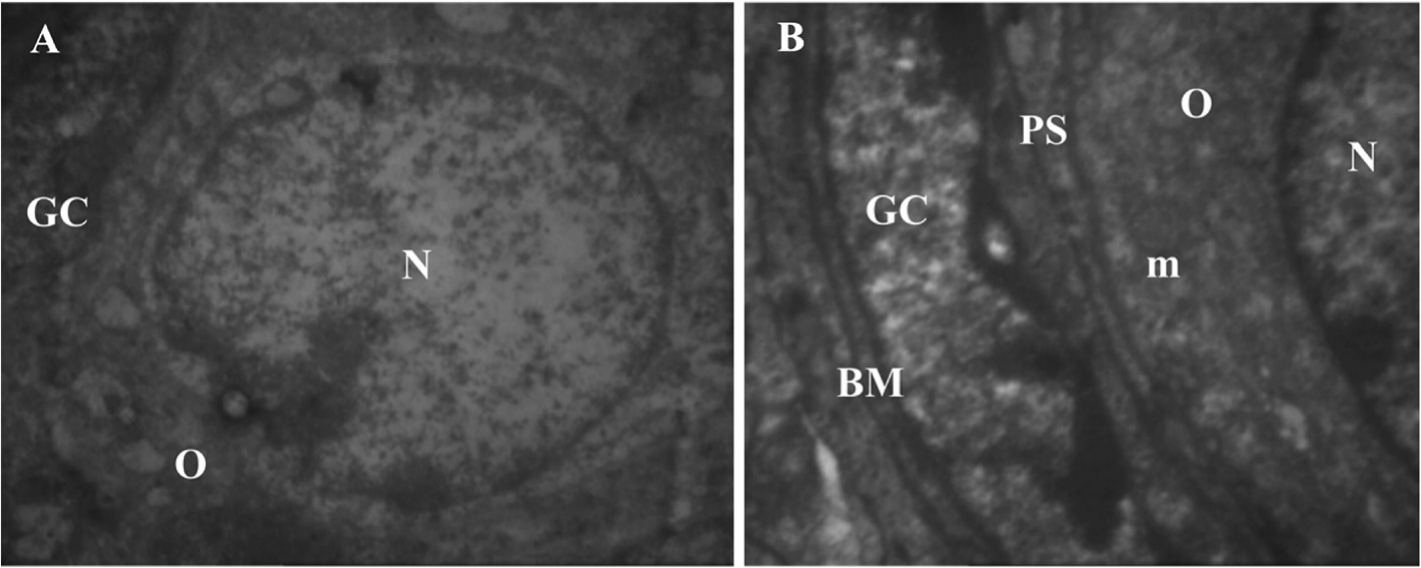
**Photomicrograph of primordial or early primary follicle intermediary follicle in the CV7 group showing the oocyte O encircled by one layer of both flattened and cuboidal granulosa cells GC.** Note the slightly centric nucleus N, a homogeneous basement membrane BM, normal mitochondria m and a narrow perivitelline space PS to form zona pellucid, **A**; TEM: ×4646, **B**; TEM: ×6000.

During histological analysis, morphologically normal follicles were characterized by a round or oval oocyte, presenting a well-delimited nucleus with uncondensed chromatin, surrounded by healthy granulosa cells closely juxtaposed to the oocyte (Figure 2A). The morphology of secondary follicles were well preserved in the CV7 and control group Also the cytoplasm of the secondary follicles and oocytes was clear and normal and all the granulose layers and theca interna and externa were intact and firmly attached to the related basement membranes (Figure 1A and I).

However in two group, some cryoinjury was observed that characterized by the disruption of intercellular contacts among innermost granulosa cells and the oocyte (Figure 1A and I). But other vitrified groups (CV1-6) showed more sign of degeneration and cryoinjury in secondary follicles (Figure 1B-H).

Moreover, in secondary follicles of CV1-6 groups were observed shrinkage and picnosis of the oocyte and granulosa cells, detachment of the follicle from the surrounding stroma, cytoplasmic retraction and numerous cytoplasmic vacuoles (Figure 1B-H).

In all treatments, there was no difference between the percentages of normal primordial, primary and secondary follicles of toxicity tests and vitrification groups (P > 0.05).

When ovarian tissue were vitrified in double cryoprotectants (EG plus DMSO), the percentages of secondary follicles whit an intact nucleus and cytoplasm was higher than in ovaries exposed to single cryoprotectants (alone EG or DMSO).

### Ultrastructural analysis of ovarian tissue

The ultrastructure of the ovarian tissue was well preserved in CV7 concentration and it was very similar to the non-vitrified group. Also there was no evidence of subcellular alterations in ovaries were vitrified in CV7 solution. All ovarian tissue vitrified in single cryoprotectant showed a poor ultrastructure. Indeed, compared to oocyte, follicular granulosa cells showed a good ultrastracture.

#### Ultrastructure of primordial follicle

The ovoid or spherical oocyte of the primordial follicles was surrounded by flattened granulosa cells. A large number of vesicles with a central position nucleus were observed in ooplasm (Figure 2A and B).

After vitrifying/thawing in double cryoprotectant, most organelles in granulosa cells and oocyte well preserved. Most mitochondria had normal cristae and a dens matrix. The smooth and rough endoplasm reticulum and golgi complex were the most prominent organelles and the cellular membranes of oocyte and granulosa cells were in close connection (Figure 2B).

In the frozen-thawed ovaries in single cryoprotectant, in some cases the mitochondria elongated and their cristae disappeard.

#### Ultrastructure of primary follicle

One or two cuboidal cells surrounded the oocyte of primary follicles. The ooplasma with spherical nucleus and the granulose cells had a large amount of organelle was very similar to that observed in the primordial follicles. The perivitelline space between cuboidal cells was filled by dens material to form zona pellucid (Figure 2B). In some cases such as single cryoprotectant solution, some mitochondria swollen and their cristae had disappeared and gap junctions were observed between oocyte and granulosa cell membranes.

#### Ultrastructure of secondary follicles

**Figure 3:**
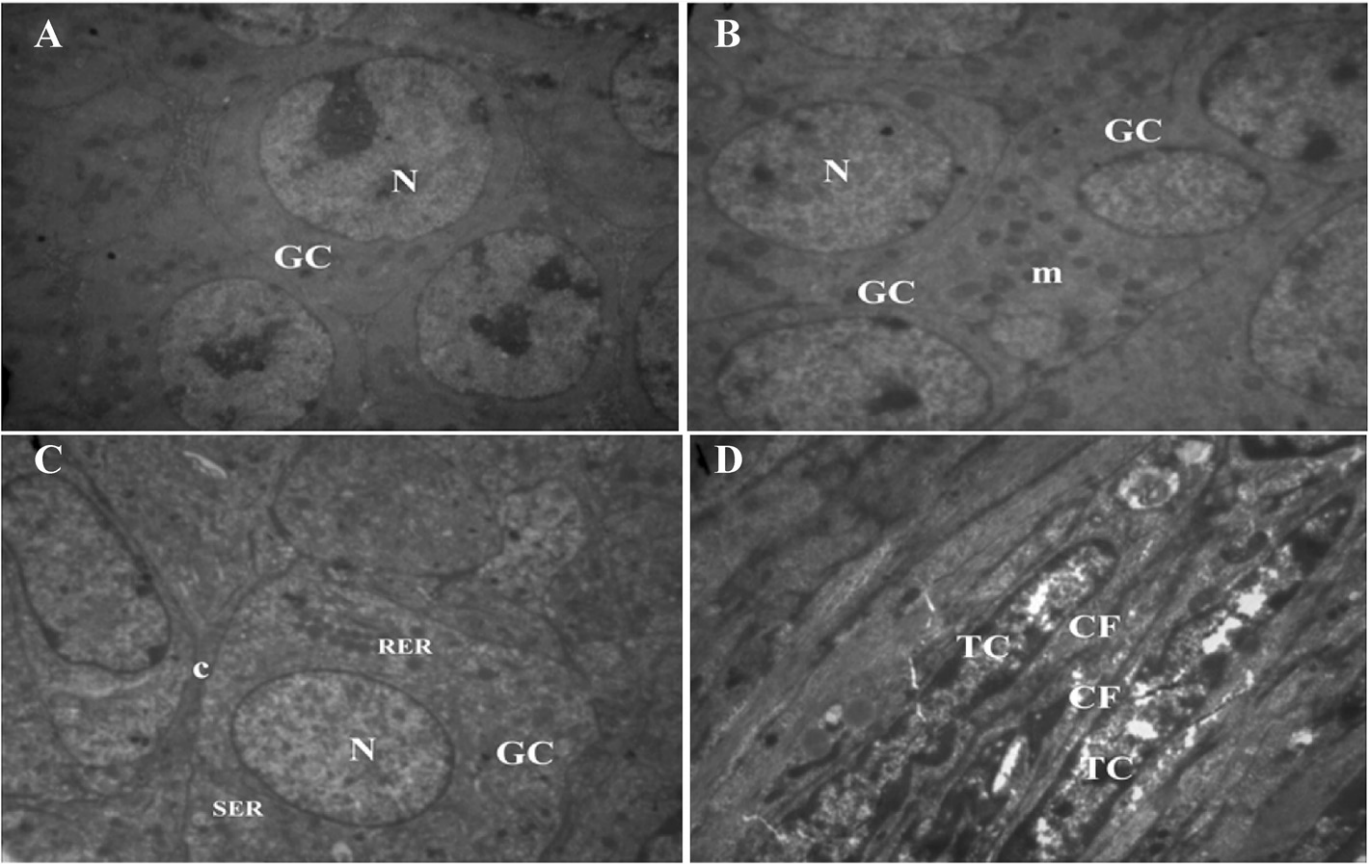
**The electron micrograph of granulosa cells of secondary follicles. A)** non-vitrified ovarian tissue, **B**, **C)** vitrified in double cryoprotectant, TEM: ×2156, **D)** theca cell of vitrified ovarian tissue in double cryoprotectant, TEM: ×3597. The intact contact c between granulosa cells GC is seen. The nucleus N and cytoplasmic organelles, mitochondria m, rough and smooth endoplasmic reticulum SER and RER are well defined. Theca cells TC in vitrified ovaries by double cryoprotectant contained collagen fibers CF is shown **D**.

The oocytes were surrounded by two or three layers of granulosa cells in secondary follicles. The fresh follicles were surrounded by a continuous basement membrane, which was tightly attached to the ovarian stroma (Figure 3A). The zona pellucida was fully developed and forming a thick layer around the oocyte and were usually associated with erect microvilli. The follicular cytoplasm and ooplasm exhibit highly variable numbers of vesicles, a well-developed Golgi complex and both smooth and rough endoplasmic reticulum and round mitochondria with continuous membranes and normal cristae were still more abundant (Figure 3A).

**Figure 4:**
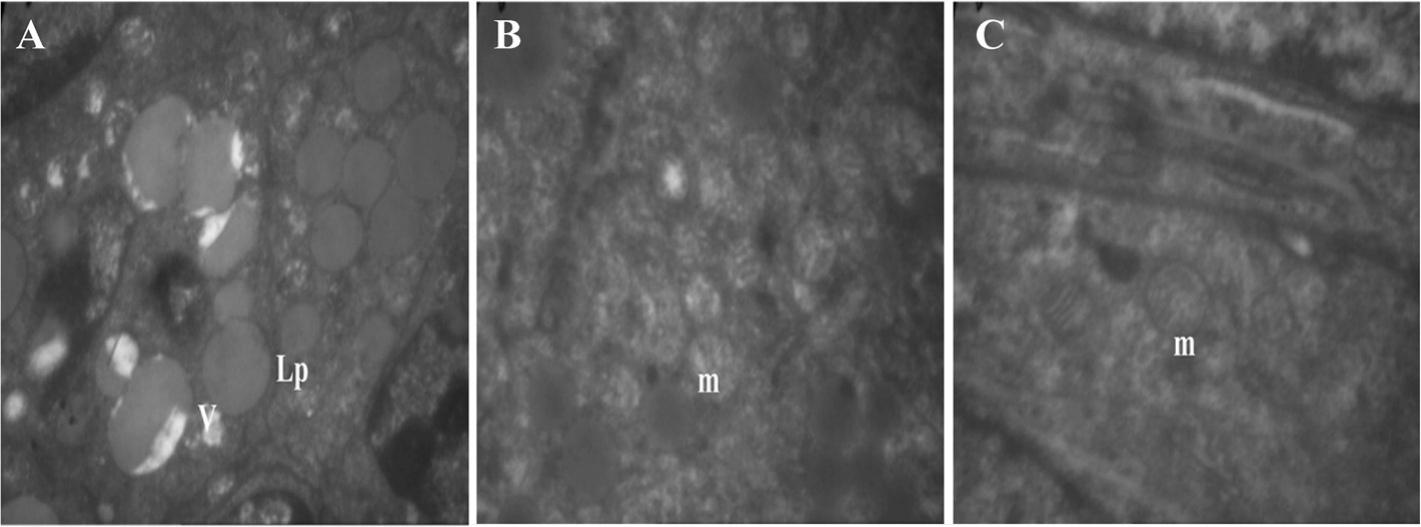
**The electron micrograph of granulosa cells of a secondary follicle. A** and **B)** vitrified ovaries in single cryoprotectant, **C)** vitrified ovaries in double cryoprotectant, showing arrangement of mithochondria m, numerous lipid droplets Lp, vacuole V and atypical or irregularly shaped mithochondria with longitudinally oriented cristae were found in CV1-4 groups or single cryoprotectant, TEM: ×10000. B; numerous spherical mithochondria with continuous membranes and normal cristae were obserced in CV6, 7 groups or double cryoprotectant, **A**; TEM: ×4646, **B** and **C**; TEM: ×10000.

The ultrastractural analysis revealed that secondary follicles of ovaries frozen in CV7 group had morphologically normal similar to those preserves in non vitrified (control) ovaries and the integrity of cell organelles was well preserved (Figure 3B and C, Figure 4C).

**Figure 5:**
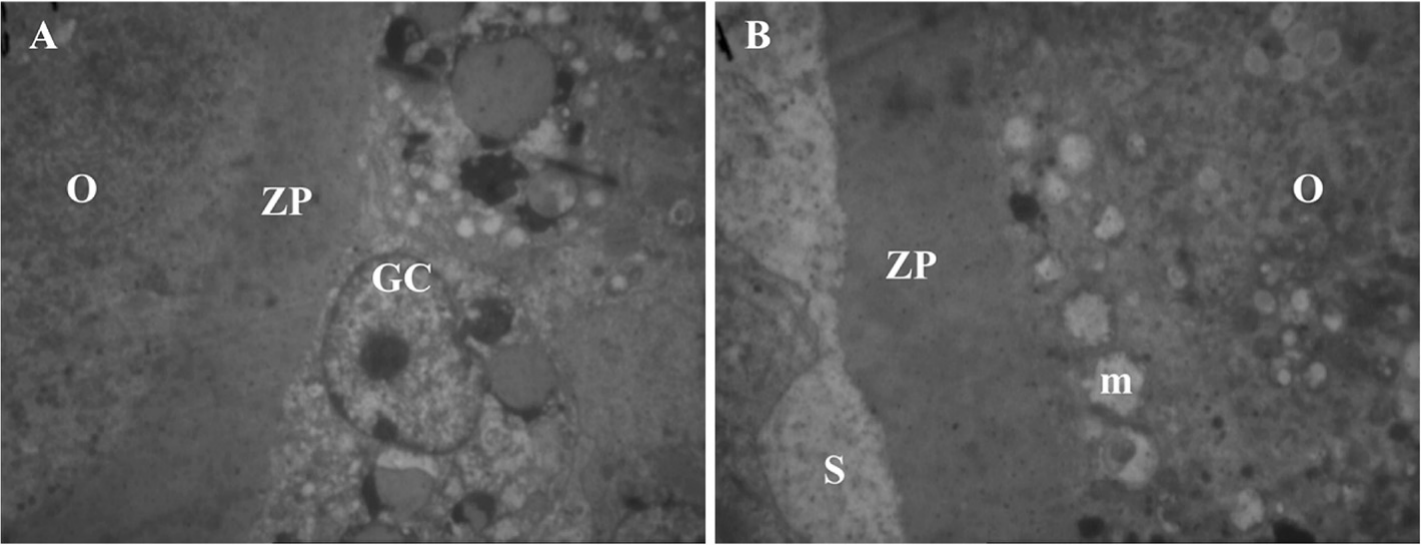
**Transmission electron microscopy images of the secondary follicle from single cryoprotectants.** The zona pellucid ZP was fully developed and forming a thick layer around the oocyte O, the oocyte had swollen mitochondria m and their crista had disappeared. Many wider spaces S observed between oocyte and granulosa cells GC. **A)** TEM: ×1670, **B)** TEM: ×2784.

After vitrifying/thawing in single cryoprotectant, the cytoplasm organelles were greatly changed in shape and distribution. The oocyte had most vacuole with nuclear shrinkage and elongated mitochondria with a few cristae (Figure 4A and B). In granulose cells, the mitochondria swollen and cristae disappeared. And also, these cells exhibited irregularly-shaped nuclei, irregular distribution of cytoplasmic organelles and vacuolated space (Figure 4A and B). Also wider space observed between oocyte and granulose cells or around of zona pellucida (Figure 5A and B).

## Discussion

We showed that the morphological integrity and ultrastructure of follicles of the ovarian tissue after vitrification with ascending concentration EG + DMSO CV7 group was significantly higher than other groups and ovarian tissue do not undergo osmotic shock after vitrification. This good result may be due to sufficient permeation of the cryoprotectants into the ovarian tissue and equilibration with stepwise cryopreservation can improve the success of cryopreserving follicles in various stages of development.

Vitrification is a useful technique which has recently been applied for cryopersavation of ovarian tissues because this method has minimal change in morphology and ultrastructure [11]. A disadvantage of the vitrification procedure, however, is the necessity of high cryoprotectant concentrations, which can cause cell damage because of their toxicity.

This adverse effects can be limited by cryopreservation strategy such as the use of a combination of vitrification solutions, each at a relatively low concentration, and therefore promoting dehydration by the increasing stepwise of solution and the inclusion of non-permeating solution in the vitrifying mixture [12-14]. Therefore, the main challenge is to achieve the optimum composition for a cryoprotectant with low biologic toxicity level which helps to vitrificaton cooling.

The results of previous study, reported that vitrification of mouse ovarian tissue using a combination of EG and sucrose achieving better results than using only EG [13,15,16]. The use of macromalcule such as disaccharides in cryopersavation method could help to prevent ice crystal formation and facilitate cryoprotectant penetration.

In this study, two type of degeneration was observed after vitrifying/thawing of ovarian tissue. The most predominant degeneration type was characterized by the disruption of intercellular contacts among innermost granulosa cells and the oocyte. Communication between oocyte and granulosa cells is necessary for endocytotic way and to the zona pellucida formation [17]. This type of degeneration was observed most often in secondary follicles of all vitrified groups, although observed in CV7 group at very low rates.

The other type of degeneration was characterized by shrinkage of the oocyte, disarrangement of granulosa cells and cytoplasmic vacuolization. This kind of degeneration was observed with great frequency in secondary follicles of ovarian tissue vitrified in single cryoprotectant.

Ultrsractural assessment of tissue samples gives much more detailed information about structural cryodamage of the cell than histological examination. Our ultrastructural studies showed that after freezing-thawing of ovaries in CV7 group, no noticeable changes occurred in the organelles of oocytes and follicular cells. The integrity of cell organelles was well preserved.

The mitochondrial organization, cytoskeletal arrangement and vacuolization are necessary key for cryopreservation assessment [18]. The mitochondria are very important to the energy producing, metabolic activation and regulating cell survival. In the ovaries vitrified by single cryoprotectant, we showed that elongated and swollen mitochondria consistently present in all the follicular developmental stages, suggesting that the mitochondria were going through an apoptosis process. Wherever Green and Reed demonstrated the mitochondria alternations are the central regulator of apoptosis [19].

The movement of mitochondria within different areas of the cell is mediated by cytoskeletal network of microtubules [20]. Reduction of cytoskeletal observed in vitrified oocyte [21]. Moreover, actin filament are the cytoskeletal core of microvili, are very sensitive to cryopreservation processes [7].

The vacuolization and lipid droplets distribution is a sign of degeneration and shrinkage of oocyte after vitrifying/thawing of ovarian tissue [22], these vacuole may represent altered mitochondria [23]. Ultrastractural analysis showed that the vitrification of ovarian tissue by single cryoprotectant characterized by lipid droplets and vacuole accumulation in the cytoplasm of secondary follicles. These alternations could be due to inadequate dehydration or concentration of cryoprotectant agents, resulted to the ice formation and damaged the organelle of granulosa cells and oocyte.

Our histological and ultrastractural analysis showed that secondary follicles are susceptible to cryoprotectant than the primordial and primary follicles. In agreement with Choi J et al. [24], after vitrifying/thawing the least damage was observed in the primordial follicle due to small size, absence of the zona pellucida and less metabolically active.

## Conclusion

We suggested that the double cryoprotectant in stepwise increasing concentration used in the present study may be appropriate for ovarian tissue vitrification. This method facilitated the exchange of water by cryoprotectant, therefore protect ovarian tissue from cryodamage and can be used to improve fertility among cancer patients.
